# Size Exclusion Chromatography Method for Purification of Nicotinamide Mononucleotide (NMN) from Bacterial Cells

**DOI:** 10.1038/s41598-018-22806-8

**Published:** 2018-03-13

**Authors:** George Cătălin Marinescu, Roua-Gabriela Popescu, Anca Dinischiotu

**Affiliations:** 1 Department of Biochemistry and Molecular Biology, University of Bucharest, Bucharest, 050095 Romania; 2 Independent Research Association, Bucharest, 012416 Romania

## Abstract

Over 12% of the world’s health resources are spent on treating diabetes, as high blood glucose is the third cause of mortality worldwide. Insulin resistance is the basis of the most common form of diabetes: type 2 diabetes. Recent animal studies report successful attempts at reversing type 2 diabetes by the administering of the NAD^+^ precursor nicotinamide mononucleotide (NMN). However, the current high price of this molecule urges for more efficient and cost-effective production methods. This work proposes a method for purifying NMN by Size Exclusion Chromatography (SEC) on silica with a covalently attached coating of poly(2-hydroxyethyl aspartamide) (PolyHEA) stationary phase using an isocratic elution with a denaturing mobile phase (50 mM formic acid) from a complex molecular mixture such as a fermentation broth. The eluted peaks were identified by UV-Vis analysis and confirmed with ESI+ mass spectrometry and a HPLC reversed-phase method. The proposed SEC method is simple, patent-free, directly applicable for industrial production with a minimum scale up effort. The need for multiple chromatographic steps is eliminated and the lysate filtration and clarification steps are simplified. Substantial reduction in NMN production costs and increased purity of NMN to the level suitable for usage in humans are expected.

## Introduction

High blood glucose level is the third highest risk factor for premature mortality worldwide. The more common condition, type 2 diabetes, has increased with modern life, urbanisation, reduced physical activity and an ageing population. It affects the blood vessels, the heart, kidneys and nerves, eventually resulting in disability and premature death^1,2^. Previously, an association between insulin resistance and type 2 diabetes has been recognised^3^. Moreover, abnormal mitochondrial function was correlated with type 2 diabetes^4–8^.

In this context, gene expression and proteomics studies revealed a correlation between insulin resistance and a down-regulation of protein complexes involved in mitochondrial oxidative phosphorylation^9–11^. Lower expression of mitochondrial complexes I-IV influences the redox state of the cell, diminishing ATP production and antioxidant capacity. In all the organs of 12 months old Wistar rats, an increase of p53 acetylation, a decreased activity of nicotinamide adenine dinucleotide (NAD^+^) dependent sirtuin-1 (SIRT1) and a mild over-expression of SIRT1 was correlated with diminished NAD^+^ level and NAD^+^/NADH ratio^12^. Although NAD^+^ is recycled by the salvage pathways^13–15^, it is consumed by poly(ADP-ribose) polymerases (PARPs) and glycohydrolases (CD38 and CD157)^16^ and therefore therapeutic interventions to restore NAD^+^ level are crucial. Nicotinamide mononucleotide (NMN), a polar molecule with a molecular weight of 334 Da^17^, was proven effective as an NAD^+^ precursor in glucose intolerance studies, restoring NAD^+^ levels in mice with type 2 diabetes induced by a high fat diet or aging^18^. The nuclear NAD^+^ level modulates mitochondrial encoded gene expression and mitochondrial homoeostasis through a new pathway regulated by SIRT1^19^. NMN was also recently found useful in limiting brain injury following intracerebral hemorrhage^20^.

In the past, NMN was prepared by incubation of diphosphopyridine nucleotide with potato pyrophosphatase^21^ or from nicotinamide by extracts of acetone-powered human erythrocytes^22^. These methods produced low quantities of NMN. Nowadays NMN is obtained by microbial biotechnology techniques. To reduce the high cost of NMN and to improve on the available purity requires innovation and optimisation of the current production methods. In this study the focus was directed on the downstream part of the biotechnological process. Only one purification method for NMN, utilising of ion exchange chromatography (IEC) followed by precipitation in acetone^23^ was identified in the scientific literature. General methods for nucleotides and other small molecules purification from biological extracts proposed separation in formic acid followed by filtration, lyophilisation, and two-dimension high performance liquid chromatography (HPLC) methods: boronate-affinity and ion-pair^24^, or reversed-phase chromatography (RPC) or via high speed counter-current chromatography (HSCCC). Several analytical HPLC methods have been reported using RPC for NMN quantification on either C18 coated silica^25^ or porous graphitic carbon stationary phase^19,26^. A similar approach, but using a gradient elution on a RP XBP C18 column (100 mm × 2.1 mm, 5 μm) and positive Electrospray Ionisation (ESI+) mass spectrometry detector running in Selected Ion Monitoring mode was previously reported for quantification of a NMN related molecule, also a NAD^+^ precursor: Nicotinamide Riboside (NR)^27^.

The goal of this study was to design a simple and cost effective NMN purification method from a complex molecular mixture, such as the lysate of *E. coli* strain BL21 (DE3) pLysS genotype *E. coli* B F–dcm ompT hsdS(rB– m B–) gal λ(DE3) [pLysS Cam^r^] [pET28a-hdNadV Km^r^].

## Results

### Single step NMN chromatographic separation by SEC-HPLC

**Figure 1:**
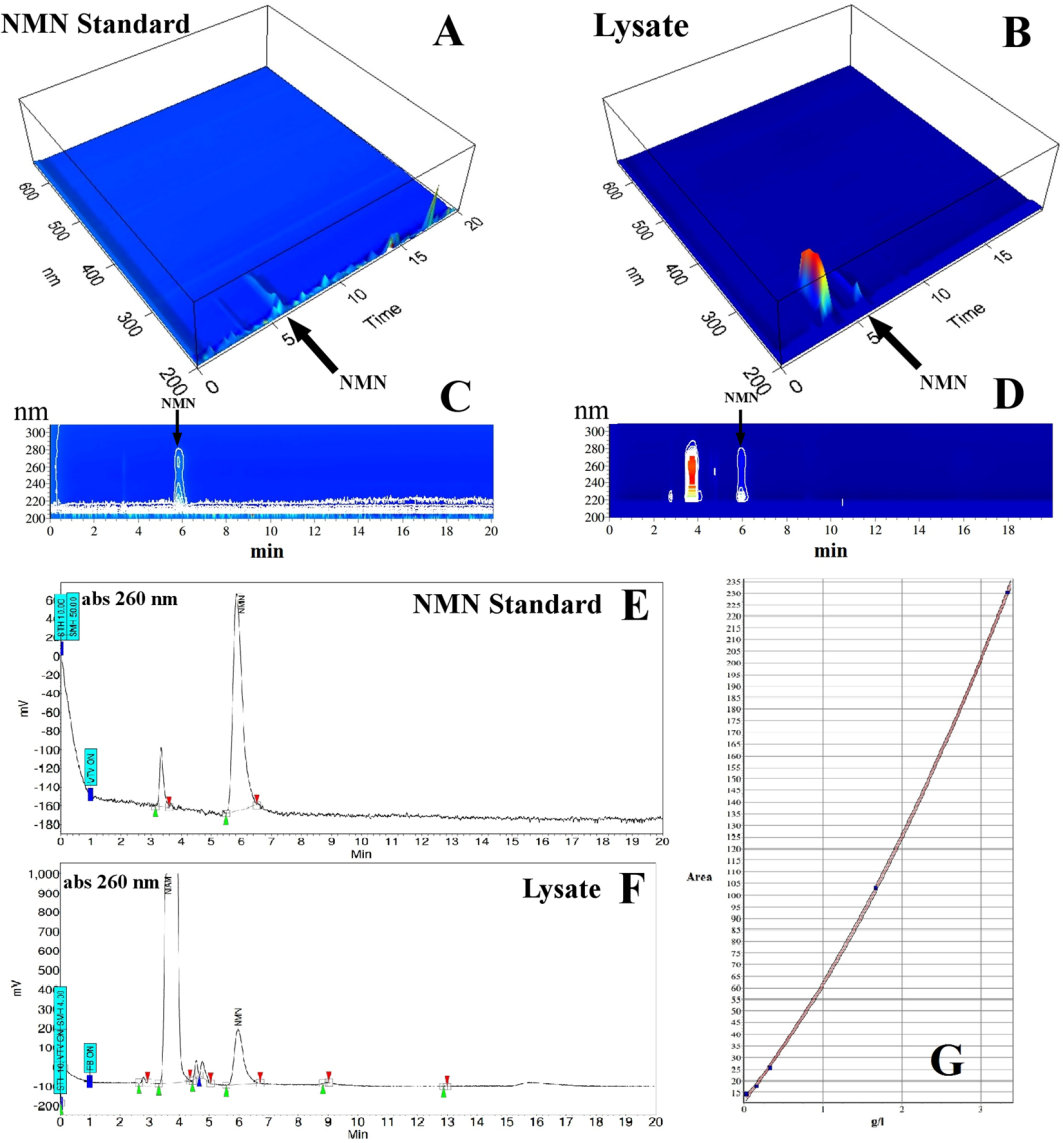
HPLC SEC separation of nicotinamide mononucleotide (NMN). The light absorption spectra (200–600 nm) (**A**,**B)**, (200–300 nm) (**C**,**D**) were recorded by the Jasco Photodiode Array (PDA) detector. (**E**,**F**) represent 260 nm chromatograms extracted from above PDA data. Mobile phase was 50 mM formic acid, with a flow rate 3 mL/min, the stationary phase was PolyHEA, column dimensions: 250 × 9.4 mm; 5 μm, 60-Å. Sample volume was 20 μL of 5 mM nicotinamide mononucleotide standard (Sigma Aldrich, however impure, as shown) (**A**,**C**,**E**), respective bacterial cell lysate (**B**,**D**,**F**). NMN elutes at 5.7 minutes and is clearly separated from other similar molecules (e.g. nicotinamide which has a similar light absorption spectrum, is more abundant in the bacterial lysate and elutes at 3.8 minutes). The second polynomial order calibration curve with a correlation coefficient of 0.99 was obtained for quantitative determination by integration of the peak area of 5 NMN samples with known concentration levels: 0.03; 0.16; 0.33; 1.67; 3.34 g/L (**G**).

The SEC-HPLC protocol was performed as explained in the Methods section employing a clarified bacterial lysate sample and standard NMN (Fig. 1A,C and E). Analysing the Photodiode Array (PDA) data led to the identification of the retention time (RT) for NMN as 6 minutes. A clear separation of NMN from other molecules of lysate is shown (Fig. 1B and D). The most abundant contaminant in the lysate had a retention time between 3.5 and 4 min (Fig. 1F) which corresponds to the impurity peak identified in the NMN standard at the same RT (Fig. 1E).

The chromatogram obtained at 260 nm was extracted from the PDA data and used for quantification by peak area integration (Fig. 1E).

For accurate NMN quantification, the calibration method described in the Methods section was used. A second order polynomial equation was obtained with the coefficient of correlation of 0.99 (Fig. 1G), covering samples with NMN concentrations from 0.03 to 3.34 g/L.

### Mass spectrometry identification of NMN

**Figure 2:**
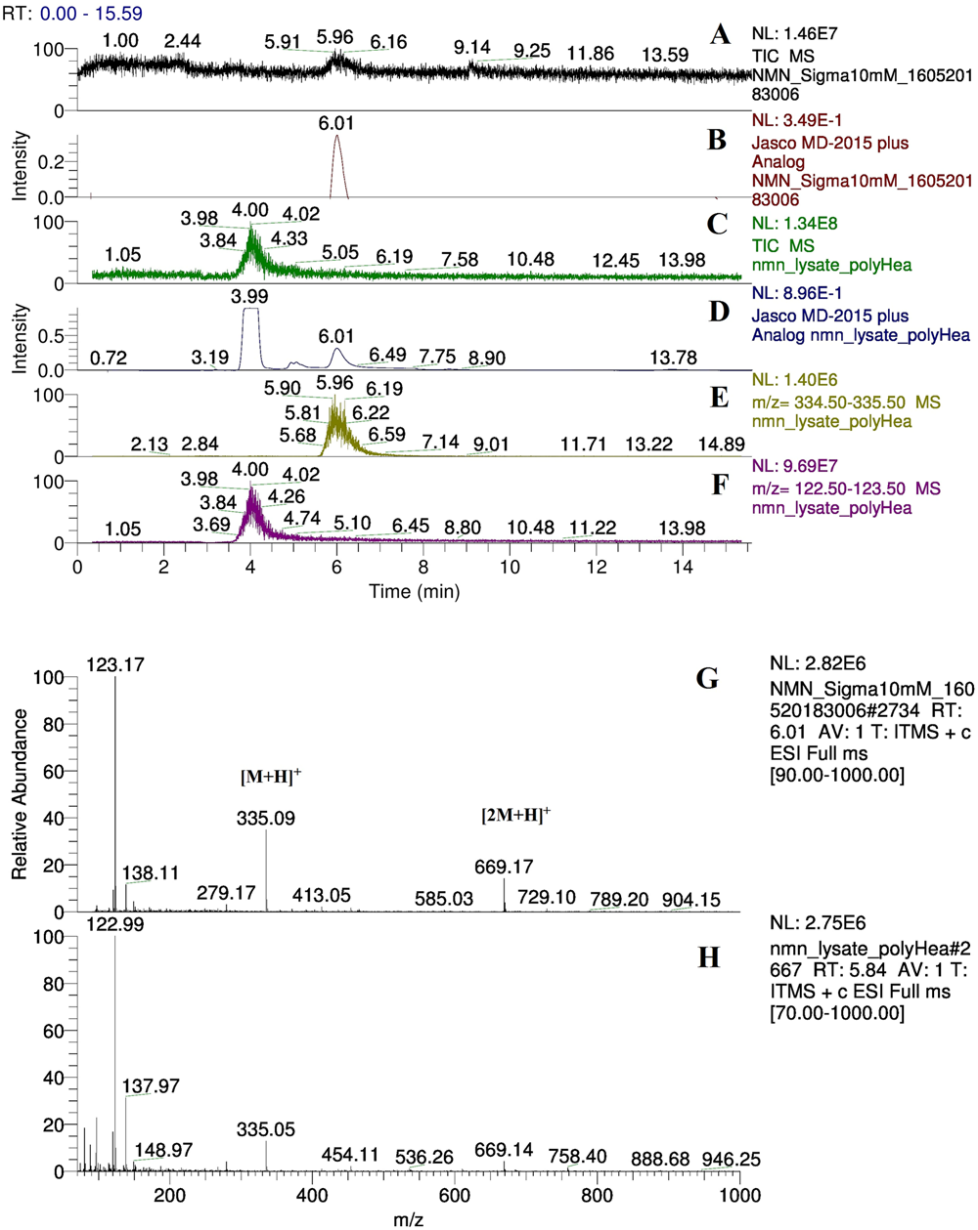
Mass spectrometry identification of nicotinamide mononucleotide (NMN). Thermo Velos Pro MS detector ESI + mode, Spray Voltage: 3 kV, Capillary temp: 375 °C; isocratic HPLC elution with 50 mM formic acid flow: 3 mL/min, PolyHEA column 250 × 9.4 mm, 5 μm, 60-Å. Sample volume: 20 μL. H-ESI-II ion source was connected parallel to the MD2015 UV-Vis detector using 1:10 post column flow splitter. Standard NMN 5 mM (Sigma Aldrich) sample elution: total Ion Current chromatogram (**A**) shows NMN eluting at 6 minutes. 260 nm Chromatogram (**B**). NMN is identified by protonated molecular ion [M + H] + (335 Da) on the MS spectrum at a retention time of 6.07 (**G**), double molecular protonated ions [2 M + H] + (669 Da) and nicotinamide (NAM) fragment protonated molecular ion (123 Da) resulted from NMN fragmentation inside the ionisation source. Bacterial lysate: Total Ion Current (**C**); HPLC UV detector 260 nm, analogue input (**D**); m/z 335 mass chromatogram showing NMN peak at RT: 5.61 (**E**); m/z 123 mass chromatogram showing NAM peak at RT: 3.65 (**F**); NMN MS spectrum showing protonated molecular ion [M + H] + (335 Da), double protonated molecular ion [2 M + H] + (669 Da) and the nicotinamide protonated molecular ion (123 Da) known to result from NMN fragmentation inside the ionisation source (**H**).

**Figure 3:**
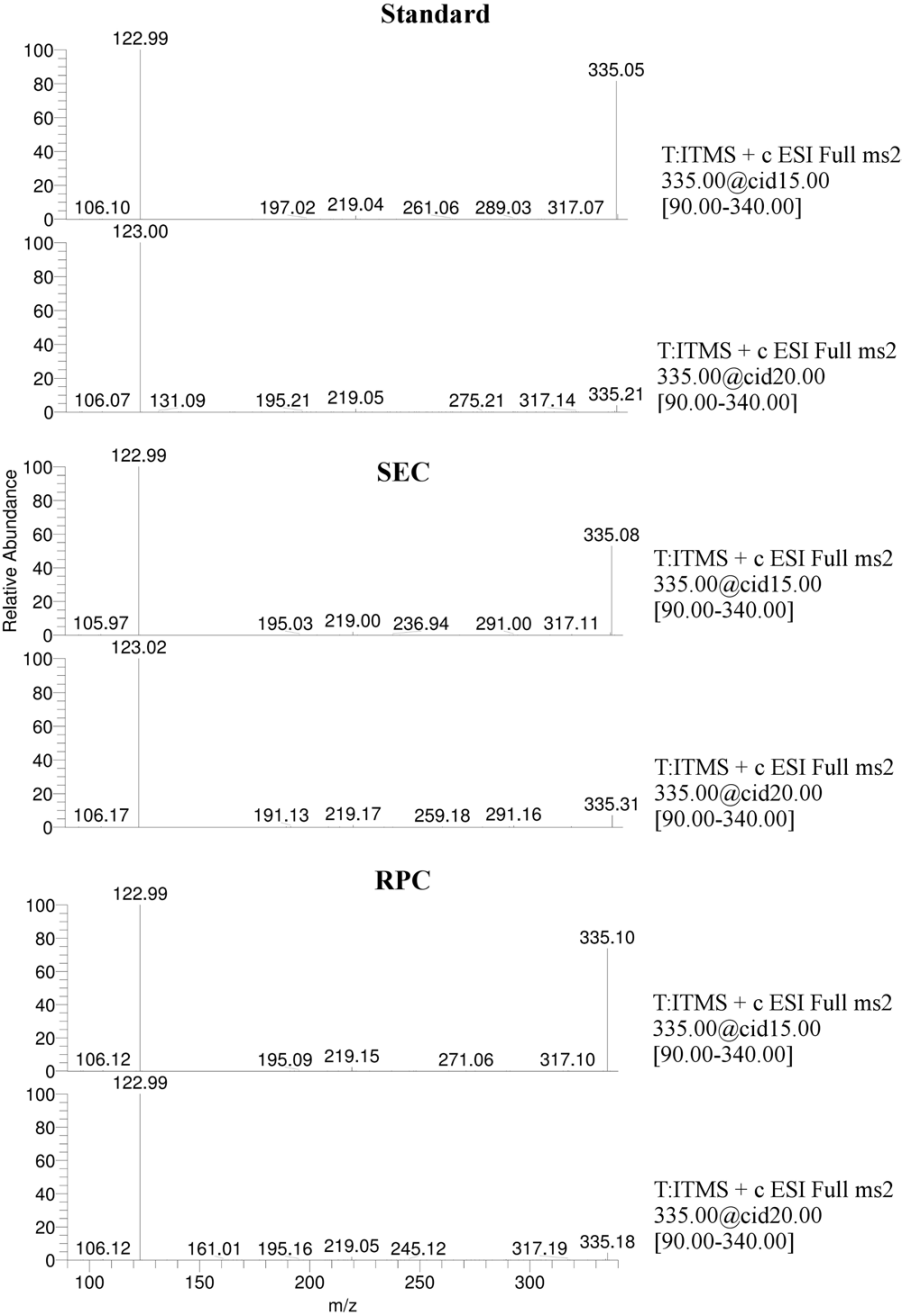
MS/MS Collision-Induced Dissociation (CID) spectral data of nicotinamide mononucleotide (NMN) from collected fractions (SEC and RPC) against standard NMN, using Thermo Velos Pro linear ion trap MS detector running in ESI+ mode, Spray Voltage: 3 kV, Capillary temperature: 375 °C, flow: 3 µL/min, H-ESI-II ion source.

Although UV-Vis light absorption data recorded by the HPLC PDA detector provide spectrum information for NMN identification, to eliminate any doubt related to the correct peak identification, a mass detector was used. The Total Ion Current (TIC) chromatogram (Fig. 2A) resulting from running the protocol described in section *Size Exclusion Chromatography High Performance Liquid Chromatography (SEC-HPLC)* (NMN standard sample, SEC protocol on PolyHEA column) shows a peak at 6 minutes, thereby correlating with the HPLC data (Fig. 2B). The MS spectrum of the NMN separated from the bacterial lysate (Fig. 2H) is similar to the spectrum of the NMN standard (Fig. 2G). The specific m/z values (shown on both standard and lysate separations) are: 335 for NMN (molecular single protonated ion [M + H]^+^); 669 for double molecular NMN single protonated ion [2 M + H]^+^; 123 for the [M + H]^+^ nicotinamide (NAM) ion, resulting from ion-source fragmentation of NMN. Selecting only m/z values between 334.5 and 335.5 (Fig. 2E) from the full MS data recorded while running the bacterial lysate separation by SEC on a PolyHEA column, a clear peak having the same RT as 260 nm chromatograms of the NMN standard (Fig. 2B) and bacterial lysate sample (Fig. 2D) is shown. The most abundant contaminant from the bacterial lysate was simultaneously identified on: TIC chromatogram (Fig. 2C), 260 nm chromatogram (Fig. 2D) and the MS chromatogram of the ions having an m/z between 122.5 and 123.5 (Fig. 2F) at a retention time of 4 min. The molecular identity of collected NMN fractions was confirmed by comparison against standard NMN MS/MS spectra (Fig. 3).

### Purity evaluation of NMN separated by SEC-HPLC using reversed-phase HPLC

**Figure 4:**
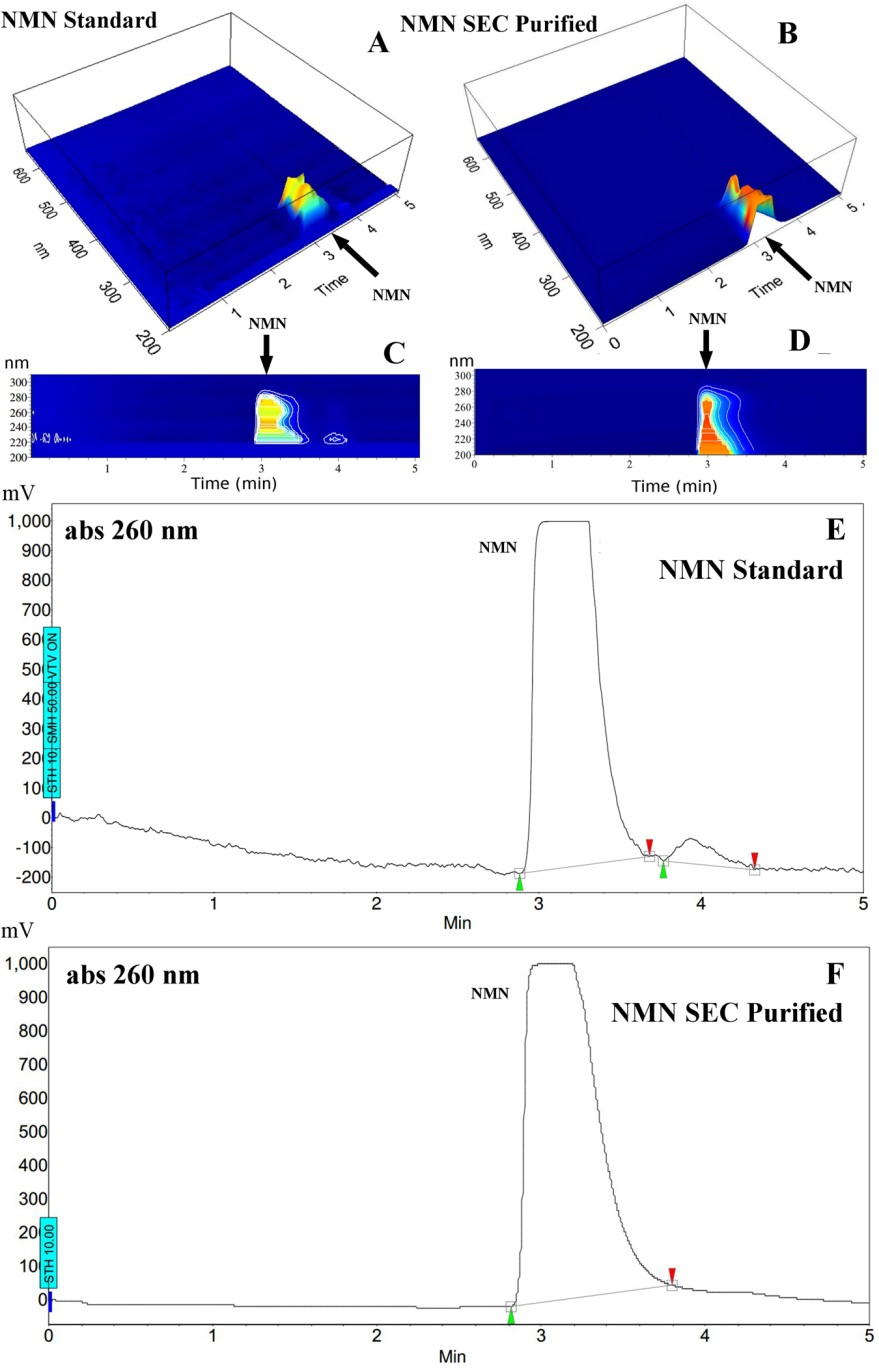
Purity evaluation of nicotinamide mononucleotide (NMN) separated by SEC PolyHEA using reversed-phase HPLC (C18). The light absorption spectrum recorded by the Jasco MD2015 UV-Vis Photodiode Array (PDA) detector during HPLC isocratic elution, NMN standard Sigma (however, impure as shown) (**A**,**C**) versus NMN purified from bacterial lysate by SEC PolyHEA (**B**,**D**). HPLC Chromatogram 260 nm isocratic elution of NMN standard Sigma 95% purity (**E**) versus NMN purified from the bacterial lysate by SEC PolyHEA (**F**). Mobile phase: 10% acetonitrile, flow rate 0.9 mL/min, column: Genesis C18 James Column 250 × 4.6 mm, 4 µm, 120 Å, injected sample: 20 μL. 10 mM NMN, starts eluting at 3 minutes.

The NMN RT using the RP-HPLC protocol was 3 minutes and the purity was determined as a percentage of the NMN peak area relative to a total detected peaks area of 260 nm chromatogram (Table 1). On the recorded 3D PDA absorption spectrum (Fig. 4A) there was no detectable signal above 280 nm therefore a plane view of the interval between 200 and 300 nm was generated. This result highlights a contaminant eluting at RT 4 min (Fig. 4C) which is clearly represented on the 260 nm chromatogram (Fig. 4E). The 3D PDA absorption spectrum of the NMN obtained from the bacterial lysate by the proposed SEC method (as shown in section *Mass spectrometry (MS)*) has no detectable impurity (Fig. 4B,D and F).

**Table 1:** Nicotinamide mononucleotide (NMN) standard (Sigma) Reversed-Phase HPLC peak detection and integration results.

Name	Retention time [min]	Quantity [%Area]	Area [mAU Min]
NMN	3.0	94.03	711.4
NAM	3.8	5.97	45.1

### Endotoxin content in NMN fractions

**Figure 5:**
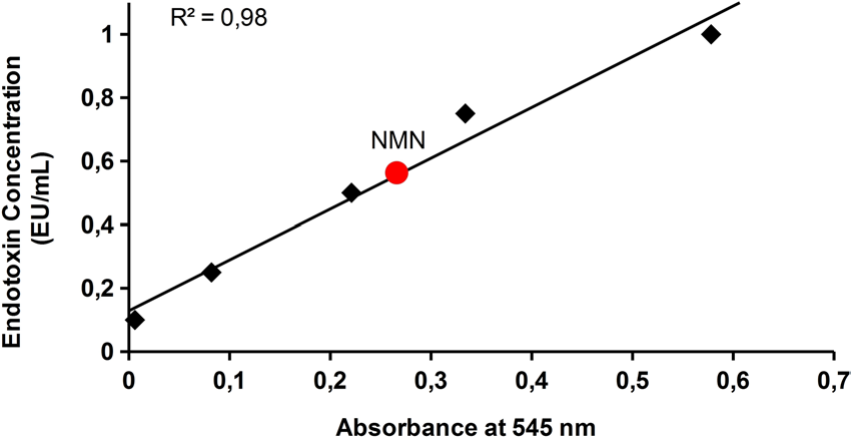
Endotoxin detection in NMN fractions by the LAL chromogenic method. The concentration of NMN sample was 1 mM.

The endotoxin level in the NMN fractions purified by SEC method was 0.51 EU/mL. This is below the calculated acceptable parenteral administration limit (1.18 EU/mL) (Fig. 5).

## Discussion

Our attempt to reproduce the previously reported ion exchange method^23^ of NMN separation from the bacterial lysate did not produce the expected results, probably because of the high nicotinamide (NAM) content of our bacterial lysate, as growing media was supplemented with 1% NAM as substrate for Nicotinamide phosphoribosyl transferase (Nampt). The mass chromatogram of ions with an m/z between 122.5 and 123.5 (Fig. 2F) corresponding to the known m/z value for NAM [M + H] + ion correlates with the RT of the most abundant impurity from the lysate (Fig. 2D). NAM and NMN co-eluted in the Dowex Cl^−^ Ion Exchange Chromatography (IEC) column and co-precipitated in acetone together with high amounts of salts (data not shown). As previously reported, IEC optimisation is a time consuming and expensive process^28^, thus efforts were spent on identification of a simpler method.

Often used for analytical purposes, RP-chromatography (RPC) was not the most desirable method for separating hydrophilic molecules because they are not well retained by the stationary phase^29^. Previously, RPC used for NMN quantification by Triple Quadrupole LC/MS/MS^25^ provided excellent analytical quantitative data. However, the results were obtained by RPC separation and selective data acquisition (multiple reaction monitoring) of the m/z values corresponding to the molecules of interest including NMN, but ignoring other possible co-eluting contaminants. This is adequate for analytical quantification but not suitable for preparative purposes. Selected-ion monitoring (SIM) filters out all information on the co-eluting molecules, and was therefore inadequate for preparative purposes. Although the further purification of the NMN standard by RPC was successful (Fig. 4C and E), the small RT difference between the two peaks limited the preparative usage of the method, while higher column load widened the peaks and decreased resolution. Since the most abundant impurity in our bacterial lysate was NAM (Figs 1F and 2D,F) and it had a similar RT with the main impurity from the NMN standard (Fig. 1E) on the proposed SEC method, it was thus concluded that the detected impurity in the NMN standard was also NAM. Therefore, the unknown peak (RT = 4 min) on the RP elution (Fig. 4C and E) of the NMN standard could be NAM. Both NAM and NMN peaks resulted by RP separation were wider than the peaks resulting from the proposed SEC method, generating a lower NMN concentration in the collected fraction and a higher likelihood of co-eluted contaminants.

The most interesting and newest RPC methods providing good RT and resolution for NMN separation use porous graphitic carbon^19,26^. The main drawback of this technique is the high price tag of this material which makes its usage prohibitive for preparative applications. A previously reported method for separation of nucleotides from whole-cell extract by formic acid extraction, filtration followed by a two-step chromatographic process: boronate-affinity and ion-pair^24^, facilitated a good separation for NMN. This, however, had the disadvantage of the two-step chromatographic process, that are more expensive compared to our proposed single step SEC.

The key of the proposed SEC separation is the stationary phase: silica with a covalently attached coating of poly(2-hydroxyethyl aspartamide) (PolyHEA). When using a non-denaturing mobile phase, the hydrogen bonds between adjacent polymer chains of the coating make the coating impermeable. When a denaturing mobile phase (like 50 mM formic acid) is used, the chaotropic agent prefers the stationary phase instead of water to form hydrogen bonds. The increased steric radius of the PolyHEA coating might occlude the 60 Å pore, but by disrupting the hydration layer the available pore volume increases and the space between polymer chains becomes accessible to the mobile phase. This distance now represents the effective pore diameter^30^. When a non-denaturing mobile phase is used with a 60 Å pore PolyHEA column, the separation range is 60–10000 Da (according to manufacturer’s manual). Using the denaturing mobile phase, the separation range shifts to 20–600 Da, which makes it suitable for separation of NMN (334 Da) from other small solutes. This is the only SEC stationary phase (according to our knowledge) having a resolution in this range.

SEC was used for two reasons: to desalt the sample to use the electrospray ionisation mass spectrometer and to filter out the higher molecular weight compounds from the mixture. Our first intention was to collect the fraction containing NMN, to identify the co-eluting contaminants and further purify it by a second chromatographic method. It was a surprise to discover that this material made unnecessary any additional purification steps. Surprisingly, using the new method, further purification of the standard NMN (Sigma Aldrich, 95% advertised purity) was made possible as illustrated in Fig. 1E. Due to the known high NAM content of the bacterial lysate, the main contaminant in the lysate was identified as NAM, which has been determined to have a retention time of 3.5–4 minutes (Fig. 1F).

**Figure 6:**
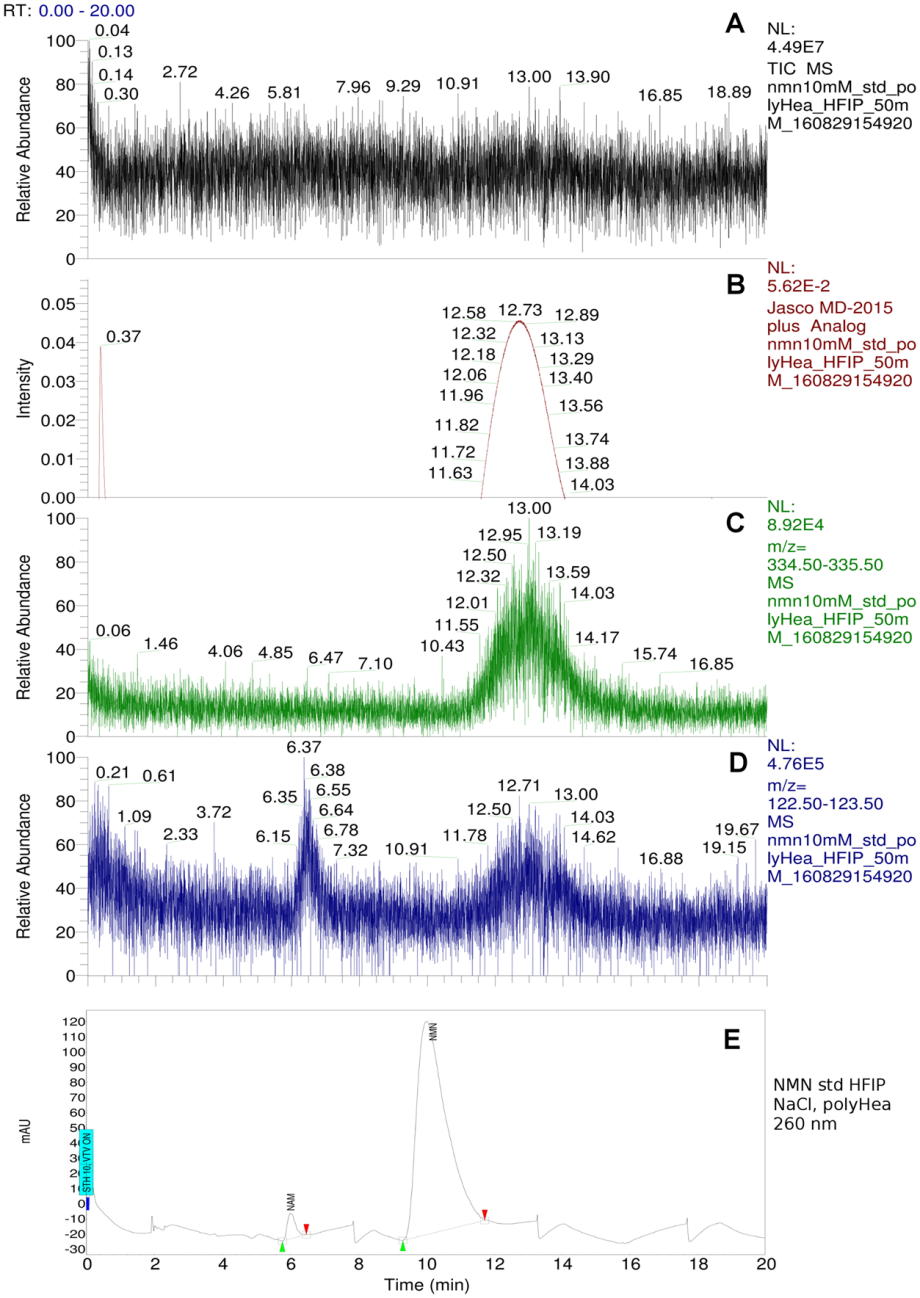
Separation of nicotinamide mononucleotide (NMN) from (NAM) using uncharged chaotrope mobile phase (50 mM 1,1,1,3,3,3-Hexafluoro-2-propanol (HFIP)), isocratic elution, flow: 3 mL/min on PolyHEA column 250 × 9.4 mm, 5 μm, 60-Å. Jasco PU2089 HPLC pump. Detectors: Thermo Velos Pro linear ion trap MS running in ESI + mode, Spray Voltage: 3 kV, Capillary temp: 375 °C, H-ESI-II ion source, connected parallel to MD2015 UV-Vis detector using 1:10 post column flow splitter. Sample volume: 20 μL of standard NMN 10 mM (Sigma Aldrich). Total Ion Current (**A**); 260 nm absorption chromatogram (**B**); 335 Da ions (**C**); 123 Da ions (**D**); The same elution conditions but mobile phase supplemented with 200 mM NaCl, to eliminate the electrostatic repulsion effect, 260 nm chromatogram UV-Vis detector only (**E**).

The data generated revealed that the elution order between NAM (122 Da) and NMN (334 Da) was surprisingly reversed. According to the SEC principle, NMN should elute first, having a greater molecular weight, suggesting that some other physical interactions occur^31^. Electrostatic charge might be the cause of the observed reversed elution order, as the titration curve of the PolyHEA material showed that at a pH below 4.4 the material behaves as having a net positive charge, and above 4.4 it behaves as though having a negative charge^30^. With 50 mM formic acid as the mobile phase, the pH was 2.0, therefore the coating net positive charge might cause electrostatic repulsion of NAM (pKa = 3.63)^32^ which also has a net positive charge at pH 2.0. The NMN phosphate group might have formed an electrostatic attraction with the coating. To verify this hypothesis, the mobile phase was initially replaced with an uncharged chaotrope i.e. 1,1,1,3,3,3-Hexafluoro-2-propanol (HFIP) 50 mM. This did not change the elution order, but increased RT for both NAM and NMN (Fig. 6). To eliminate any electrostatic effect, 200 mM NaCl was added to the mobile phase. In these conditions, RT of NAM was the same while NMN RT was slightly shorter, without changing the elution order (Fig. 6E). This suggested that although electrostatic interactions determine coating repulsion for both NAM and (unexpectedly) NMN, they are not the cause of the reversed elution order, as eliminating them does not restore the theoretical SEC elution order. No further investigations on the matter were performed, as it does not serve the purpose of this study.

Although an ultracentrifugation step was performed, that was convenient for small scale processing during the development of the method, for higher scale processing, filtration should be used instead. Moreover, the same filtration method and equipment could be used for both cell separation and lysate clarification. Another advantage of the SEC method presented in this study is that salt molecules (known to be harmful for the mass spectrometry electrospray ionisation source) have a longer RT so the flow can be diverted to waste after elution of the useful fractions, thereby protecting the equipment. Thus, the method applied also performs desalting without the need for any additional processing steps. As previously reported^24^ the formic acid from the collected NMN fraction is simply removed by lyophilisation.

Mass spectrometry confirmed NMN peaks identified by the UV-Vis spectrum. Specific MS (Fig. 2) and MS/MS spectra (Fig. 3), consistent with previously reported data^25–27^ were identified in both standard and lysate, increasing certainty of the identity of the obtained product. Considering the molecular diversity of the bacterial lysate, the theoretical possibility to have an impurity with similar size but lower concentration compared to NMN still existed. This type of impurity could hypothetically have the same RT as NMN on the proposed SEC method. As NMN was more abundant, running the MS detector in Full Scan mode could also fail to detect this hypothetical impurity. A typical example is the lack of detection for a 123 m/z peak caused by the fragmentation of NMN in the ionisation source at RT = 6 min during the SEC lysate separation (Fig. 2F). Although the m/z = 123 signal is present (Fig. 2H), it is completely eclipsed on the m/z 122.5–123.5 ion chromatogram by the strong NAM peak at RT = 4. Therefore, the product resulting from SEC was evaluated by the second HPLC method based on a completely different separation principle (reversed-phase). During the RP separation, no impurity in the product collected from SEC was observed (Fig. 4B,D,F). Only the NMN UV absorption spectrum was observed which supported the results of this study that SEC alone separates NMN from the bacterial lysate.

Although the pharmaceutical formulation was beyond the scope of this study, the endotoxin level had to be controlled in case of parenteral administrated drugs. Its concentration was 0.51 EU/mL, below the accepted limit of 1.18 EU/mL. This resulted in a strong suspicion that the detected endotoxin level in the NMN fractions was caused by the HPLC solvents used, which were not labelled as endotoxin free. This hypothesis was supported by the determined endotoxin level in the laboratory water used in the HPLC solvents which was 1.52 EU/mL, three times higher than in the NMN sample. Having a molecular mass greater than 10 kDa, lipopolysaccharides (LPS) resulting from the biotechnological processes are large molecules, which sediment during the ultracentrifugation step. In the less probable situation when they are still present in the clarified lysate, SEC is a known method for their elimination^33,34^, and that is the reason for the lower endotoxin level detected in our NMN sample compared with the HPLC solvents used. Evidently, an even lower endotoxin level can be obtained by using endotoxin free solvents. In the case of oral administration, endotoxin contamination is not a concern, as they do not pass through an intestinal barrier^35^.

Growing evidence of anti-ageing and anti-diabetic effects of nicotinamide mononucleotide (NMN) and the current high price tag of this promising molecule were the two key facts motivating us to accomplish this work. The proposed method of NMN purification by Size Exclusion HPLC (SEC) on silica with a covalently attached coating of poly(2-hydroxyethyl aspartamide) (PolyHEA) stationary phase using isocratic elution with denaturing mobile phase (50 mM formic acid) is the first proposed single step chromatographic method for NMN purification from a complex molecular mixture such as a bacterial lysate. The method is simple, patent-free, directly applicable for industrial production with a minimum scale up effort, with bulk material being commercially available to prepare higher capacity columns. It not only eliminates the need for multiple chromatographic steps, but also simplifies the lysate filtration and clarification process, since SEC is not influenced by higher molecular mass compounds which are nonetheless not retained by the column. Therefore, the proposed purification method should substantially contribute to reduction of NMN production costs while also increasing the purity to the level suitable for usage in humans.

## Methods

### NMN Purification Workflow

**Figure 7:**
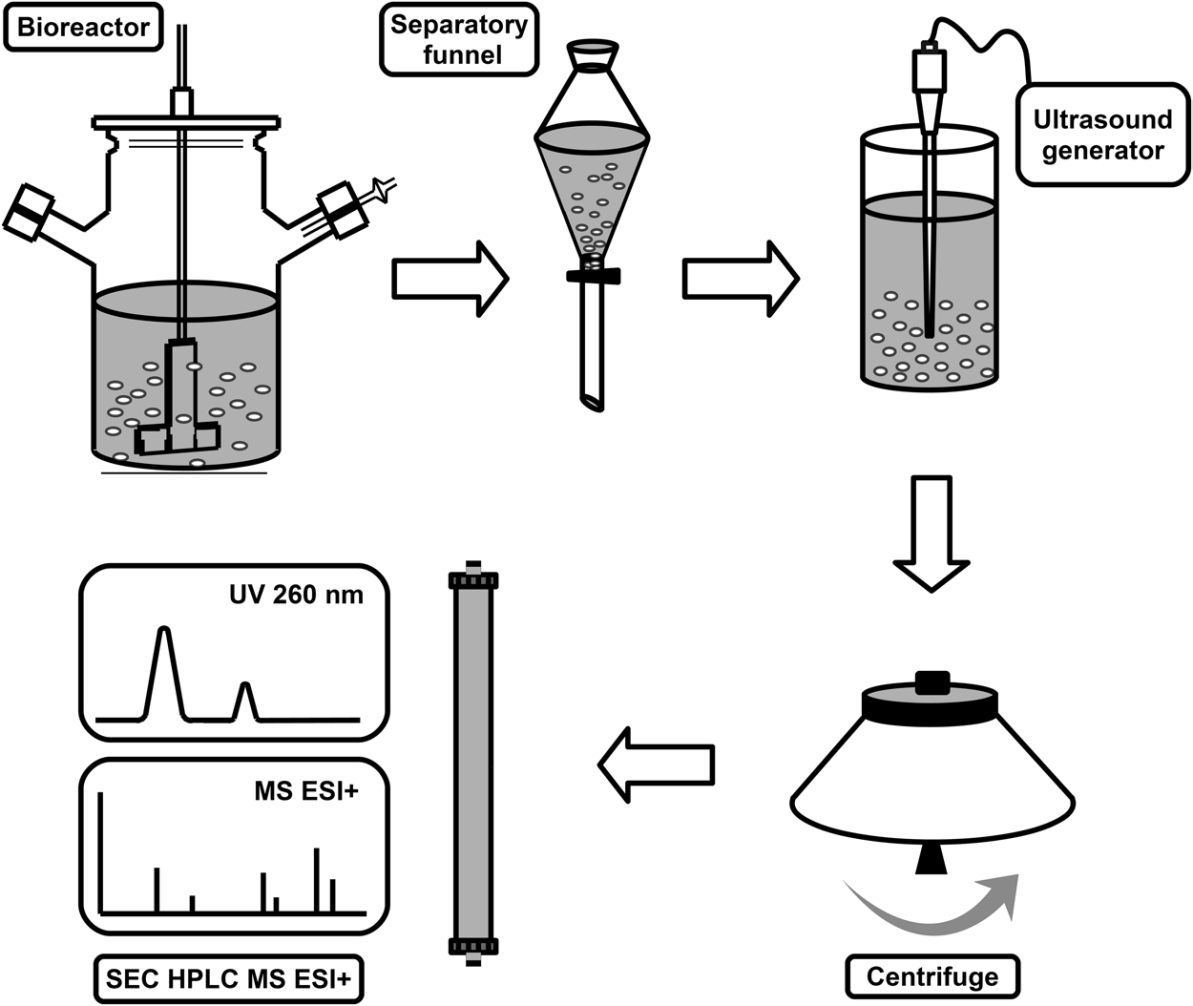
The schematic diagram for the proposed nicotinamide mononucleotide (NMN) purification process. Bacterial cells are grown in the bioreactor, the media containing bacteria is pumped into a separating funnel and stored at 4 °C. After 24 hours, the sedimented bacterial cells are collected, cell membranes are disrupted by ultrasound on ice, cell debris and macromolecules are separated by ultracentrifugation and discarded, NMN is finally separated by a single step size exclusion chromatography (SEC) on a PolyHEA column eluted with 50 mM formic acid.

The process was designed following the classical biotechnological process workflow from the bacterial broth to the pure final product^31,36^. The bacterial cells were grown in a 10 L bioreactor and were separated from the media using a 10 L separatory funnel. The cell membranes were disrupted using a sonicator cell disruptor. The cell lysate was clarified by centrifugation and NMN was purified by a single step size exclusion chromatography technique (Fig. 7).

### Strains, Media, Growth Conditions and Separation of bacterial cells

An overnight culture of *E. coli* strain BL21(DE3)pLysS genotype *E. coli* B F–dcm ompT hsdS(rB– m B–) gal λ(DE3) [pLysS Cam^r^] [pET28a-hdNadV Km^r^] (a gift from Independent Research Association, Bucharest - 012416, Romania), 50 mL, grown in a shaking incubator at 250 rpm was used as inoculum for a 10 L bench-top bioreactor culture. This strain is carrying a plasmid (pET28a-hdNadV, Addgene ID #83362) expressing a gene (nadV) from *Haemophilus ducreyi* coding for Nampt catalysing the direct production of NMN from nicotinamide (NAM). LB growing media (NaCl 10 g/L, Tryptone 10 g/L, yeast extract 5 g/L) supplemented with 1% NAM and 1% glucose was sterilised in autoclave for 20 minutes at 120 °C. Once the sterilised media cooled down below 60 °C, kanamycin was added to a final concentration of 50 µg/mL. The temperature for bacterial culture was set to 37 °C. The bioreactor was continuously measuring the optical density of the culture at 600 nm (OD600), the stirring motor speed being set to 150 rpm. Once OD600 reached 0.45, the expression of nadV was induced by adding Isopropyl-1-thio-β-D-galactopyranoside (IPTG) to a final concentration of 1 mM. When the measured OD600 of the culture reached 0.75, the media containing bacterial cells was automatically pumped into a 10 L separatory funnel (supplied by Adrian Sistem SRL) and left at 10 °C for 24 h. The funnel tap was then slowly opened, and 100 mL of cells rich media were collected.

### Extraction and Clarification

The collected cells were evenly distributed in five 50 mL Falcon Conical Centrifuge Tubes and the cell membranes were disrupted by sonication at 20 kHz on ice in 3 cycles of 30 seconds separated by 30 seconds pause using a sonicator cell disruptor model W185F (Heat Systems-Ultrasonic Inc.). The lysate was ultracentrifuged using the Beckman Optima LE-80 K for 3 hours at 500,000 *g* at 4 °C. The supernatant was carefully collected and used for NMN purification by High Performance Liquid Chromatography (HPLC).

### High Performance Liquid Chromatography (HPLC)

#### Size Exclusion Chromatography High Performance Liquid Chromatography (SEC-HPLC)

Samples of 20 μL were consequently poured on the SEC column.

The Jasco HPLC PU-2089 pump and Rheodyne 20 μL manual injection loop were connected to 250 × 9.4 mm; 5 μm, 60 Å PolyHEA column (silica with a covalently attached coating of poly(2-hydroxyethyl aspartamide) purchased from PolyLC INC (Item# 259HY05006). Post column, a 10:1 ratio flow splitter was used to connect a Jasco UV-Vis Photodiode Array (PDA) MD-2015 on the high flow side, respective of the Heated Electrospray Ionisation (H-ESI-II) from Thermo Scientific Velos Pro Linear Ion Trap Mass Spectrometer on the low flow side. Between the flow splitter and the ionisation source, the flow was passed through the mass spectrometer divert/inject valve as specified in the Thermo Velos Pro user manual. Jasco LC-NETII-ADC controlled by Chrompass 1.8.6.1. software was used to run the LC System while the LTQ Tune Plus 2.7 and Xcalibur 2.2. were used for the mass spectrometry detector and acquisition process. The Jasco LC system was configured to generate the acquisition start signal for the MS to ensure reproducible retention times (RT) were achieved on both data systems. Analog channel 1 from the LC system was connected to the 1 V analogue input of the mass spectrometer. Mass spectrometry grade water (39253 Fluka LC-MS CHROMASOLV) and formic acid (94318 Fluka) were purchased from Sigma-Aldrich as well as other chemicals and reagents, unless otherwise stated. For NMN SEC separation, the isocratic solvent A: 50 mM formic acid was used at a flow rate of 3 mL/min for 20 minutes. The UV-Vis light absorption spectrum (200–640 nm) was continuously recorded. The light absorption chromatogram at 260 nm was recorded by both Chrompass and Xcalibur software.

External standard NMN (Sigma N3501-25MG) solutions were prepared for 5 different NMN concentrations (0.03, 0.16, 0.33, 1.67 and 3.34 g/L), of which 20 µL samples were consecutively eluted by SEC, in the ascending order of concentration. The NMN peak areas of the 260 nm chromatograms were then used to generate the calibration curve by regression^37^ for NMN quantification in Chrompass Software (Fig. 1G).

NMN fractions were collected each time, mixed together, lyophilised and stored at −80 °C.

#### Reversed-Phase High Performance Liquid Chromatography (RP-HPLC)

The NMN purified by SEC was hydrated in 200 µL deionised water, 20 µL of the resulting solution was injected in the HPLC sample loop and eluted following the same protocol, previously described.

The Jasco HPLC PU-2089 pump and Rheodyne 20 µL manual injection loop were connected to the Genesis C18 James Column 250 × 4.6 mm; 4 µm; 120 Å column and the Jasco UV-Vis Photodiode Array (PDA) MD-2015 was connected to the column output. The HPLC system was controlled by Jasco LC-NETII-ADC and Chrompass 1.8.6.1. software. A volume of 20 µL of 10 mM NMN (Sigma) standard solution was also injected. Isocratic elution with 10% acetonitrile at a flow rate of 0.9 mL/min for 5 minutes was used. The light absorption spectrum (220–640 nm) was recorded and a 260 nm chromatogram was generated.

### Mass spectrometry (MS)

The mass spectrometer connected to the HPLC system as described in the *Size Exclusion Chromatography High Performance Liquid Chromatography (SEC-HPLC)* section was running in ESI + mode, Spray Voltage: 3 kV, Capillary Temperature was 375 °C. The full m/z range from 70 to 1000 Da was scanned. Prior to sample injection, the mass spectrometer divert valve was switched to the inject position, the MS data acquisition was started from the LTQ Tune software with the “waiting for contact closure” option. Thus, the start acquisition signal was generated by the HPLC injection. After 15 minutes of acquisition, the MS valve was switched to the divert position to protect the instrument from salts. Using Xcalibur software, the total ion current chromatogram and the signal intensity corresponding to m/z between 334.5 and 335.5 Da respective 122.5 and 123.5 Da were extracted from full MS raw data. The 1 V analogue input of the MS was continuously recording a 260 nm absorption data sent by the Jasco MD-2015 plus detector. MS/MS spectra (m/z: 90–340) for NMN containing fractions were obtained by Collision-Induced Dissociation (CID, energy values: 15 and 20) of single charged protonated ion (m/z = 335).

### Limulus amoebocyte lysate (LAL) assay

The lipopolysaccharides (LPS) content from the SEC-purified NMN fractions were determined by the Limulus amoebocyte assay chromogenic method following the kit manufacturer’s instructions (ToxinSensor^TM^ Chromogenic LAL Endotoxin Assay Kit, GenScript, Piscataway, USA) which had a sensitivity between 0.005 and 1 endotoxin units per millilitre (EU/mL). The human equivalent dose (HED) of NMN was calculated as being 40.5 mg/kg by the methodology presented by Nair & Jacob^38^, starting from doses used without any observed adverse effect in animal studies^18,39,40^. The concentration of NMN sample was 1 mM which is above the minimum valid concentration (MVC) determined as 0.038 mg/mL^41^. At a daily perfusable dose of 2.4 g NMN in 250 mL isotonic solution the endotoxin calculated limit is 1.18 EU/mL^42^.

## References

[1] International Diabetes Federation, IDF Diabetes Atlas. Available from http://www.diabetesatlas.org/ (2015).

[2] World Health Organization (WHO), Global Report on Diabetes. http://www.who.int/diabetes/en/ (2016).

[3] Taylor R (2012). Insulin resistance and type 2 diabetes. Diabetes.

[4] Martin SD, McGee SL (2014). The role of mitochondria in the aetiology of insulin resistance and type 2 diabetes. Biochim. Biophys. Acta.

[5] Montgomery MK, Turner N (2015). Mitochondrial dysfunction and insulin resistance: an update. Endocr. Connect..

[6] Petersen KF, Dufour S, Befroy D, Garcia R, Shulman GI (2004). Impaired mitochondrial activity in the insulin-resistant offspring of patients with type 2 diabetes. N. Engl. J. Med..

[7] Szendroedi J, Phielix E, Roden M (2011). The role of mitochondria in insulin resistance and type 2 diabetes mellitus. Nat. Rev. Endocrinol..

[8] Ennequin G (2017). Neuregulin 1 improves complex 2-mediated mitochondrial respiration in skeletal muscle of healthy and diabetic mice. Sci. Rep..

[9] Skov V, Glintborg D, Knudsen S, Jensen T (2007). Expression of Nuclear-Encoded Genes Involved in Mitochondrial Oxidative Metabolism in Skeletal Muscle of Insulin-Resistant Women With Polycystic Ovary Syndrome. Diabetes.

[10] Kim J-A, Wei Y, Sowers JR (2008). Role of mitochondrial dysfunction in insulin resistance. Circ. Res..

[11] Burkart AM (2016). Insulin Resistance in Human iPS Cells Reduces Mitochondrial Size and Function. Sci. Rep..

[12] Braidy N (2011). Age related changes in NAD+ metabolism oxidative stress and sirt1 activity in wistar rats. PLoS One.

[13] Houtkooper RH, Cantó C, Wanders RJ, Auwerx J (2010). The secret life of NAD+: An old metabolite controlling new metabolic signaling pathways. Endocr. Rev..

[14] Sporty J, Lin SJ, Kato M, Ognibene T, Stewart B (2009). Characterization of chromosomal integration sites for heterologous gene expression in Saccharomyces cerevisiae. Yeast.

[15] Aragonès G (2016). Dietary proanthocyanidins boost hepatic NAD+ metabolism and SIRT1 expression and activity in a dose-dependent manner in healthy rats. Sci. Rep..

[16] Mouchiroud L, Houtkooper RH, Auwerx J (2013). NAD+ metabolism: a therapeutic target for age-related metabolic disease. Crit. Rev. Biochem. Mol. Biol..

[17] National Center for Biotechnology Information, PubChem Compound Database; CID = 14180. https://pubchem.ncbi.nlm.nih.gov/compound/14180 (2017).

[18] Yoshino J, Mills KF, Yoon MJ, Imai SI (2011). Nicotinamide mononucleotide, a key NAD+ intermediate, treats the pathophysiology of diet- and age-induced diabetes in mice. Cell Metab..

[19] Zhang T (2009). Enzymes in the NAD+ salvage pathway regulate SIRT1 activity at target gene promoters. J. Biol. Chem..

[20] Wei, C., Kong, Y., Li, G., Guan, Y. & Wang, P. Nicotinamide mononucleotide attenuates brain injury after intracerebral hemorrhage by activating Nrf2/HO-1 signaling pathway. *Sci. Rep*. 1–13, 10.1038/s41598-017-00851-z (2017). 10.1038/s41598-017-00851-z PMC5429727 28386082

[21] Plauf GWE, Plaut KA (1953). A Method for the Purifkation of Nicotinamide Mononucleotide. Arch. Biochem. Biophys..

[22] Preiss J, Handler P (1957). Enzymatic synthesis of nicotinamide mononucleotide. J. Biol. Chem..

[23] Armarego, W. L. F. & Chai, C. L. L. In *Purification of Laboratory Chemicals* (eds Armarego, W. L. F. & Chai, C. L. L.) 690 (Elsevier, 2009).

[24] Payne SH, Ames BN (1982). A procedure for rapid extraction and high-pressure liquid chromatographic separation of the nucleotides and other small molecules from bacterial cells. Anal. Biochem..

[25] Yamada K, Hara N, Shibata T, Osago H, Tsuchiya M (2006). The simultaneous measurement of nicotinamide adenine dinucleotide and related compounds by liquid chromatography/electrospray ionization tandem mass spectrometry. Anal. Biochem..

[26] Trammell SA, Brenner C (2013). Targeted, LCMS-based Metabolomics for Quantitative Measurement of NAD(+) Metabolites. Comput. Struct. Biotechnol. J..

[27] Dong W-R (2014). New function for Escherichia coli xanthosine phophorylase (xapA): genetic and biochemical evidences on its participation in NAD(+) salvage from nicotinamide. BMC Microbiol..

[28] Swadesh, J. K. Ion exchange chromatography in *HPLC Practical and* Industrial *Applications* (ed. Swadesh, J. K.), 213–287 (CRC Press LLC, 2001).

[29] Larson, J. R., Tingstad, J. E., Swadesh, J. K. Chapter one Introduction in *HPLC Practical and Industrial Applications* (ed. Swadesh, J. K.), 2–49 (CRC Press LLC, 2001).

[30] Alpert, A. J. Chapter 8. Size exclusion high-performance liquid chromatography of small solutes in *Column Handbook for Size Exclusion Chromatography* (ed. Wu, C. S.) 249–266 (Academic Press Inc., 1999).

[31] Cannell R (1998). How to approach the isolation of a natural product. Nat. Prod. Isol..

[32] Drugbank, Nicotinamide https://www.drugbank.ca/drugs/DB02701 (2017).

[33] London AS, Mackay K, Lihon M, He Y, Alabi BR (2014). Gel filtration chromatography as a method for removing bacterial endotoxin from antibody preparations. Biotechnol. Prog..

[34] Ongkudon CM, Chew JH, Liu B, Danquah MK (2012). Chromatographic Removal of Endotoxins: A Bioprocess Engineer’s Perspective. ISRN Chromatogr..

[35] Dawson, M. E. Endotoxin testing in *Pharmaceutical Dosage Forms - Parenteral Medications Third Edition: Volume 2: Facility Design, Sterilization and Processing* (eds. Nema, S. & Ludwig, J. D.) 146–187 (CRC Press, 2010).

[36] Merchuk, J. C. & Gluz, M. Bioreactors, Air-Lift Reactors in *Encyclopedia of Bioprocess Technology: Fermentation, Biocatalysis and Bioseparation* (eds. Flickinger, M. C. & Drew, S. W.) 320–353 (John Wiley & Sons, Inc., 1999).

[37] Snyder, E. & Kirkland, J. J. Quantitation (including trace analysis) in *Practical HPLC Method Development* (ed. Kruse, H. P.) 655 (John Wiley & Sons, 1997).

[38] Nair A, Jacob S (2016). A simple practice guide for dose conversion between animals and human. J. Basic Clin. Pharm..

[39] Gomes AP (2013). Declining NAD+ induces a pseudohypoxic state disrupting nuclear-mitochondrial communication during aging. Cell.

[40] Yamamoto, T. *et al*. Nicotinamide mononucleotide, an intermediate of NAD+ synthesis, protects the heart from ischemia and reperfusion. *PLoS One* **9** (2014). 10.1371/journal.pone.0098972 PMC4048236 24905194

[41] Dawson ME (1995). Maximum Valid Dilution and Minimum Valid Concentration. LAL Updat..

[42] Dawson ME (1995). Endotoxin Limits. LAL Updat..

